# Di-μ-aqua-bis­[diaqua­bis(thio­cyanato-κ*N*)iron(II)] 4-(4-chloro­phen­yl)-1,2,4-triazole hexa­solvate

**DOI:** 10.1107/S1600536808019326

**Published:** 2008-07-05

**Authors:** Xiuhua Li, Ya Zuo

**Affiliations:** a College of Chemistry, Chifeng University, Inner Mongolia 024000, People’s Republic of China; b College of Science, Inner Mongolia Agricultural University, Inner Mongolia 010018, People’s Republic of China

## Abstract

The title complex, [Fe_2_(NCS)_4_(H_2_O)_6_]·6C_8_H_6_ClN_3_, comprises two distorted octa­hedral iron(II) centers straddling a crystallographic inversion center and bridged by two aqua O atoms to form a quadrilateral core. The aqua O atom of the core is involved in hydrogen bonds with the triazole N atoms of the solvent mol­ecules, generating one-dimensional ladder motifs, and three inter­molecular C—H⋯S hydrogen bonds, forming a three-dimensional hydrogen-bonding network.

## Related literature

For related literature, see: Hsu *et al.* (1999 ▶); MacMurdo *et al.* (2000 ▶); Nordlund & Eklund (1993 ▶); Sazinsky *et al.* (2004 ▶); Stubbe & Van der Donk (1998 ▶); Yoon *et al.* (2004 ▶); Zheng *et al.* (1999 ▶).
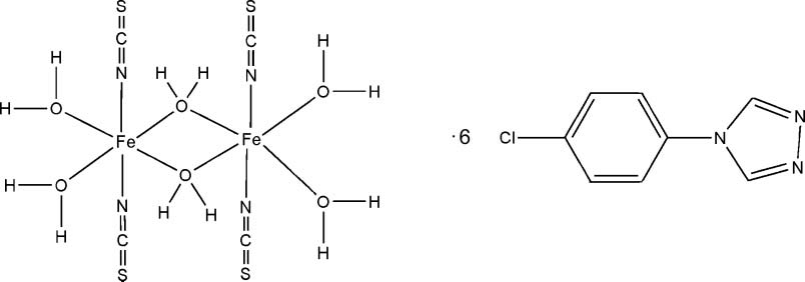


## Experimental

### 

#### Crystal data

[Fe_2_(NCS)_4_(H_2_O)_6_]·6C_8_H_6_ClN_3_
*M* *_r_* = 1529.76
Triclinic, 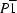

*a* = 7.944 (3) Å
*b* = 11.085 (5) Å
*c* = 19.912 (10) Å
α = 105.613 (10)°
β = 97.750 (10)°
γ = 97.932 (7)°
*V* = 1645.1 (12) Å^3^
*Z* = 1
Mo *K*α radiation
μ = 0.88 mm^−1^
*T* = 298 (2) K
0.25 × 0.21 × 0.17 mm

#### Data collection

Bruker SMART CCD area-detector diffractometer
Absorption correction: multi-scan (*SADABS*; Sheldrick, 1996 ▶) *T*_min_ = 0.810, *T*_max_ = 0.865
8642 measured reflections
5705 independent reflections
2903 reflections with *I* > 2σ(*I*)
*R*_int_ = 0.032

#### Refinement

*R*[*F* ^2^ > 2σ(*F* ^2^)] = 0.044
*wR*(*F* ^2^) = 0.076
*S* = 0.77
5705 reflections
439 parameters
9 restraints
H atoms treated by a mixture of independent and constrained refinement
Δρ_max_ = 0.25 e Å^−3^
Δρ_min_ = −0.24 e Å^−3^

### 

Data collection: *SMART* (Bruker, 1998 ▶); cell refinement: *SAINT* (Bruker, 1998 ▶); data reduction: *SAINT*; program(s) used to solve structure: *SHELXS97* (Sheldrick, 2008 ▶); program(s) used to refine structure: *SHELXL97* (Sheldrick, 2008 ▶); molecular graphics: *SHELXTL* (Sheldrick, 2008 ▶); software used to prepare material for publication: *SHELXTL* and *PLATON* (Spek, 2003 ▶).

## Supplementary Material

Crystal structure: contains datablocks I, global. DOI: 10.1107/S1600536808019326/si2093sup1.cif

Structure factors: contains datablocks I. DOI: 10.1107/S1600536808019326/si2093Isup2.hkl

Additional supplementary materials:  crystallographic information; 3D view; checkCIF report

## Figures and Tables

**Table:** 

Fe1—N1	2.086 (3)
Fe1—O2	2.100 (2)
Fe1—O3	2.102 (3)
Fe1—N2	2.107 (3)
Fe1—O1^i^	2.264 (3)
Fe1—O1	2.281 (2)

**Table:** 

N1—Fe1—O2	90.22 (11)
N1—Fe1—O3	89.68 (12)
O2—Fe1—O3	101.01 (10)
N1—Fe1—N2	178.33 (12)
O2—Fe1—O1^i^	89.04 (10)
O3—Fe1—O1^i^	169.95 (9)
N2—Fe1—O1^i^	91.32 (11)
O1^i^—Fe1—O1	78.36 (9)
Fe1^i^—O1—Fe1	101.64 (9)

Symmetry code: (i) 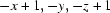.

**Table 2:** Hydrogen-bond geometry (Å, °)

*D*—H⋯*A*	*D*—H	H⋯*A*	*D*⋯*A*	*D*—H⋯*A*
O1—H1⋯N10^ii^	0.87 (3)	1.97 (3)	2.827 (4)	170 (3)
O1—H2⋯N9	0.88 (3)	1.94 (3)	2.819 (4)	173 (3)
O2—H3⋯N7^iii^	0.88 (3)	1.98 (3)	2.866 (5)	178 (3)
O2—H4⋯N4^iv^	0.88 (2)	1.97 (3)	2.853 (4)	175 (3)
O3—H5⋯N6	0.88 (3)	1.92 (3)	2.802 (4)	174 (3)
O3—H6⋯N3^v^	0.88 (2)	1.93 (2)	2.803 (4)	172 (3)
C3—H7⋯S2^vi^	0.93	2.72	3.624 (5)	165
C22—H21⋯S2^ii^	0.93	2.87	3.736 (5)	156
C11—H13⋯S1^vii^	0.93	2.87	3.783 (5)	167

Symmetry codes: (ii) 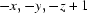; (iii) 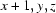; (iv) 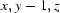; (v) 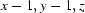; (vi) 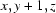; (vii) 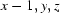.

**Fig. 1.**
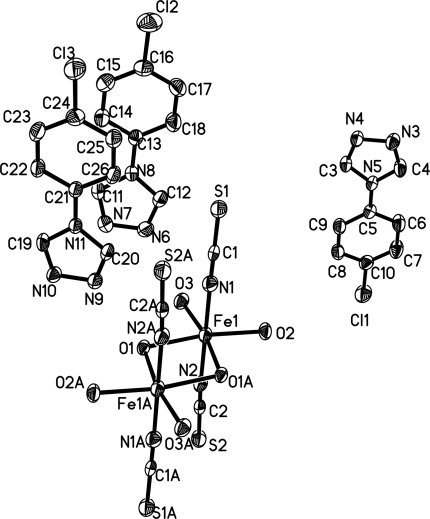
The structure of the title complex, showing 30% probability displacement ellipsoids and the atom-numbering schemes. H atoms have been omitted for clarity. Atoms of the inversion-related half-complex are indicated with A, symmetry code: (-x + 1, -y, -z + 1).

**Fig. 2.**
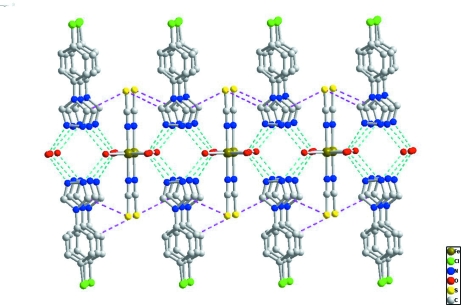
The three-dimensional structure of the title complex, the chains were drawn in different colors. Dashed lines indicate hydrogen bonds.

## supplementary crystallographic information

### Comment

The diiron unit, with a carboxylate-rich coordination environment, continue to attract considerable attention due to the enzyme catalysis activity, which occur in related multicompent dioxygen dependent enzymes, including toluene monooxygenase (Sazinsky *et al.*, 2004), the R2 subunit of ribonucleotide reductase (Stubbe & Van der Donk, 1998; Nordlund & Eklund, 1993). With the development of compounds that contained the diiron center, the structure of a series of Fe2(II,II) (MacMurdo *et al.*, 2000), Fe2(III,III) (Zheng *et al.*, 1999) and Fe2(III,IV) (Hsu *et al.*, 1999) complexes with a central 2Fe2O quadrilateral have been currently obtained. Compared with the chelating to the iron atoms with the carboxylic oxygen atoms, it is rarely reported that the quadrilateral center includes both aqueous oxygen atoms. In order to explore the furthur details of the coordinated environment of the diiron system, the title complex was synthesized. As shown in Fig. 1, the complex structure comprises two distorted octahedron iron(II) centers straddling a crystallographic inversion center bridged by two aqueous oxygen atoms to form a quadrilateral core. The separation between the iron atoms is 3.523 (2) Å, which is remarkably different from that 3.0430 (7)Å reported previously, owing to the absence of two carboxylate ligands (Yoon *et al.*, 2004). Moreover, the distance of Fe—Fe is comparatively distinguished from that diiron containing the other higher valence of iron (MacMurdo *et al.*, 2000; Zheng *et al.*, 1999; Hsu *et al.*, 1999). The bond lengths of Fe—O1 and Fe—O1a are 2.264 (3) and 2.281 (2) Å, and the angles of O1—Fe—O1a and Fe1a—O1—Fe are 78.36 (9)° and 101.64 (9)°. Each Fe(II) center resides in a six-coordinated octahedron of N_2_O_4_. On the equator plane, the center is bridged by two symmetrical O1 (water) to form the quadrilateral core with the mean distance of 2.272 (2) Å, and is connected with O2 and O3 offered by different waters as the terminal ligands with the bond lengths 2.102 (3)Å and 2.100 (2) Å. The axial positions are occupied by two N atoms from the NCS^-^ anions with the distances 2.086 (3)Å and 2.107 (3)Å to the iron core. Selected bonds and angles are listed in Table 1. As indicated in Fig.2, the classic intermolecular O—H···N H-bonds are formed between the triazol nitrogen atom supplied by the uncoordinated organic ligand 1,2,4-triazol-chloro-benzene and aquous oxygen atoms supplied by the bridging and terminal water ligands to generate a one-dimension ladder structure with the N···O separation ranged from 2.803 (2)Å to 2.866 (4) Å. Moreover, there are three weak intermolecular hydrogen bonding contacts C—H···S that form a three-dimensional network with the C···S distances between 3.624 (5) Å and 3.783 (5) Å. The details of the hydrogen bonds are shown in Table 2.

### Experimental

The compound was synthesized under hydrothermal conditions. A mixture of *L* (*L*=1,2,4-triazol-chloro-benzene) (0.3 mmol, 0.0538 g), FeSO_4_˙7H_2_O (0.1 mmol, 0.028 g), KSCN (0.2 mmol, 0.019 g) and water (10 mL) was placed in a 25 mL acid digestion bomb and heated at 433 K for two days, then equably cooled to room temperature for three days. After washed by 5 ml water for twice, green block crystals of the compound were obtained.

### Refinement

The water H atoms were located in a Fourier difference map and refined subject to an O—H restraint 0.88 (1)Å and an H···H restraint of 1.42 (2)Å. Other H atoms were allowed to ride on their parent atoms with C—H distances of 0.93 Å (*U*_iso_(H)=1.2*U*_eq_(C)). All of the non-hydrogen atoms were refined anisotropically.

### Crystal data

**Table:**

[Fe_2_(NCS)_4_(H_2_O)_6_]·6C_8_H_6_ClN_3_	*Z* = 1
*M**_r_* = 1529.76	*F*_000_ = 780
Triclinic, *P*1	*D*_x_ = 1.544 Mg m^−^^3^
*a* = 7.944 (3) Å	Mo *K*α radiation λ = 0.71073 Å
*b* = 11.085 (5) Å	Cell parameters from 1471 reflections
*c* = 19.912 (10) Å	θ = 2.5–22.0º
α = 105.613 (10)º	µ = 0.88 mm^−^^1^
β = 97.750 (10)º	*T* = 298 (2) K
γ = 97.932 (7)º	Block, green
*V* = 1645.1 (12) Å^3^	0.25 × 0.21 × 0.17 mm

### Data collection

**Table:**

Bruker SMART CCD area-detector diffractometer	5705 independent reflections
Radiation source: fine-focus sealed tube	2903 reflections with *I* > 2σ(*I*)
Monochromator: graphite	*R*_int_ = 0.032
*T* = 298(2) K	θ_max_ = 25.0º
φ and ω scans	θ_min_ = 2.4º
Absorption correction: multi-scan(SADABS; Sheldrick, 1996)	*h* = −9→9
*T*_min_ = 0.811, *T*_max_ = 0.865	*k* = −11→13
8642 measured reflections	*l* = −21→23

### Refinement

**Table:**

Refinement on *F*^2^	Secondary atom site location: difference Fourier map
Least-squares matrix: full	Hydrogen site location: inferred from neighbouring sites
*R*[*F*^2^ > 2σ(*F*^2^)] = 0.044	H atoms treated by a mixture of independent and constrained refinement
*wR*(*F*^2^) = 0.076	*w* = 1/[σ^2^(*F*_o_^2^) + (0.0191*P*)^2^] where *P* = (*F*_o_^2^ + 2*F*_c_^2^)/3
*S* = 0.77	(Δ/σ)_max_ = 0.002
5705 reflections	Δρ_max_ = 0.25 e Å^−^^3^
439 parameters	Δρ_min_ = −0.24 e Å^−^^3^
9 restraints	Extinction correction: none
Primary atom site location: structure-invariant direct methods	

### Special details

**Table:**

Geometry. All e.s.d.'s (except the e.s.d. in the dihedral angle between two l.s. planes) are estimated using the full covariance matrix. The cell e.s.d.'s are taken into account individually in the estimation of e.s.d.'s in distances, angles and torsion angles; correlations between e.s.d.'s in cell parameters are only used when they are defined by crystal symmetry. An approximate (isotropic) treatment of cell e.s.d.'s is used for estimating e.s.d.'s involving l.s. planes.
Refinement. Refinement of *F*^2^ against ALL reflections. The weighted *R*-factor *wR* and goodness of fit *S* are based on *F*^2^, conventional *R*-factors *R* are based on *F*, with *F* set to zero for negative *F*^2^. The threshold expression of *F*^2^ > σ(*F*^2^) is used only for calculating *R*-factors(gt) *etc*. and is not relevant to the choice of reflections for refinement. *R*-factors based on *F*^2^ are statistically about twice as large as those based on *F*, and *R*- factors based on ALL data will be even larger.

### Fractional atomic coordinates and isotropic or equivalent isotropic displacement parameters (Å^2^)

**Table:**

	*x*	*y*	*z*	*U*_iso_*/*U*_eq_	
Fe1	0.53640 (6)	0.12392 (5)	0.46090 (2)	0.04255 (16)	
Cl1	0.81665 (15)	0.70325 (12)	−0.09593 (6)	0.0942 (4)	
Cl2	0.41822 (16)	0.88209 (11)	0.91545 (5)	0.0932 (4)	
Cl3	0.28229 (14)	0.59403 (11)	1.00137 (5)	0.0879 (4)	
N1	0.5944 (4)	0.2571 (3)	0.56111 (15)	0.0518 (9)	
N2	0.4754 (3)	−0.0065 (3)	0.35844 (15)	0.0493 (8)	
N3	1.0886 (4)	1.1192 (3)	0.31387 (16)	0.0633 (10)	
N4	0.9153 (4)	1.0935 (3)	0.31608 (16)	0.0545 (9)	
N5	0.9562 (4)	0.9986 (3)	0.20814 (15)	0.0469 (8)	
N6	0.2192 (4)	0.4095 (3)	0.51909 (16)	0.0575 (9)	
N7	0.0498 (4)	0.3852 (3)	0.53051 (17)	0.0575 (9)	
N8	0.2018 (4)	0.5377 (3)	0.62253 (16)	0.0447 (8)	
N9	0.1490 (4)	0.1463 (3)	0.59896 (14)	0.0468 (8)	
N10	−0.0244 (4)	0.1290 (3)	0.60322 (15)	0.0461 (8)	
N11	0.1199 (3)	0.2618 (3)	0.70379 (14)	0.0395 (7)	
O1	0.3213 (3)	0.0112 (2)	0.49662 (13)	0.0402 (6)	
O2	0.7680 (3)	0.2021 (3)	0.43624 (14)	0.0524 (7)	
O3	0.3662 (3)	0.2333 (3)	0.42643 (14)	0.0517 (7)	
S1	0.67311 (13)	0.44880 (10)	0.69114 (5)	0.0643 (3)	
S2	0.40098 (12)	−0.15078 (10)	0.21581 (5)	0.0616 (3)	
C1	0.6275 (4)	0.3375 (3)	0.61526 (19)	0.0414 (9)	
C2	0.4443 (4)	−0.0668 (3)	0.29919 (19)	0.0432 (10)	
C3	0.8410 (5)	1.0220 (4)	0.2526 (2)	0.0539 (11)	
H7	0.7229	0.9906	0.2394	0.065*	
C4	1.1071 (5)	1.0625 (4)	0.2506 (2)	0.0658 (13)	
H8	1.2132	1.0651	0.2357	0.079*	
C5	0.9232 (5)	0.9260 (4)	0.13488 (19)	0.0478 (10)	
C6	1.0521 (5)	0.9321 (4)	0.0947 (2)	0.0678 (13)	
H9	1.1598	0.9828	0.1154	0.081*	
C7	1.0201 (5)	0.8626 (4)	0.0238 (2)	0.0769 (14)	
H10	1.1065	0.8654	−0.0032	0.092*	
C8	0.8611 (5)	0.7902 (4)	−0.00595 (19)	0.0603 (11)	
C9	0.7322 (5)	0.7823 (4)	0.0331 (2)	0.0647 (12)	
H11	0.6246	0.7318	0.0120	0.078*	
C10	0.7645 (5)	0.8500 (4)	0.1036 (2)	0.0633 (12)	
H12	0.6784	0.8446	0.1305	0.076*	
C11	0.0450 (5)	0.4635 (4)	0.5915 (2)	0.0558 (11)	
H13	−0.0540	0.4683	0.6115	0.067*	
C12	0.3038 (5)	0.5010 (4)	0.5745 (2)	0.0594 (11)	
H14	0.4198	0.5364	0.5802	0.071*	
C13	0.2501 (5)	0.6260 (3)	0.69190 (19)	0.0439 (9)	
C14	0.1375 (5)	0.6350 (4)	0.7392 (2)	0.0592 (11)	
H15	0.0265	0.5867	0.7250	0.071*	
C15	0.1885 (5)	0.7152 (4)	0.8074 (2)	0.0665 (12)	
H16	0.1112	0.7220	0.8389	0.080*	
C16	0.3512 (6)	0.7843 (4)	0.82879 (19)	0.0579 (11)	
C17	0.4632 (5)	0.7791 (4)	0.7819 (2)	0.0668 (13)	
H17	0.5730	0.8292	0.7963	0.080*	
C18	0.4138 (5)	0.7001 (4)	0.7134 (2)	0.0657 (12)	
H18	0.4902	0.6966	0.6817	0.079*	
C19	−0.0374 (4)	0.1979 (3)	0.66556 (19)	0.0474 (10)	
H19	−0.1410	0.2029	0.6821	0.057*	
C20	0.2307 (5)	0.2255 (3)	0.65901 (19)	0.0497 (10)	
H20	0.3497	0.2536	0.6699	0.060*	
C21	0.1591 (4)	0.3441 (3)	0.77494 (18)	0.0412 (9)	
C22	0.0297 (4)	0.3605 (3)	0.81508 (19)	0.0527 (11)	
H21	−0.0833	0.3191	0.7954	0.063*	
C23	0.0681 (5)	0.4380 (4)	0.8839 (2)	0.0591 (11)	
H22	−0.0194	0.4500	0.9104	0.071*	
C24	0.2350 (5)	0.4975 (4)	0.91359 (19)	0.0560 (11)	
C25	0.3640 (5)	0.4839 (4)	0.8742 (2)	0.0591 (11)	
H23	0.4763	0.5268	0.8941	0.071*	
C26	0.3268 (4)	0.4065 (3)	0.80535 (19)	0.0524 (10)	
H24	0.4147	0.3961	0.7790	0.063*	
H1	0.237 (3)	−0.039 (3)	0.4646 (12)	0.094 (15)*	
H2	0.276 (4)	0.058 (3)	0.5303 (14)	0.108 (18)*	
H3	0.854 (3)	0.260 (3)	0.4649 (14)	0.108 (18)*	
H4	0.809 (4)	0.170 (3)	0.3975 (10)	0.078 (15)*	
H5	0.323 (4)	0.287 (3)	0.4580 (14)	0.100 (17)*	
H6	0.279 (3)	0.192 (3)	0.3924 (12)	0.085 (15)*	

### Atomic displacement parameters (Å^2^)

**Table:**

	*U*^11^	*U*^22^	*U*^33^	*U*^12^	*U*^13^	*U*^23^
Fe1	0.0324 (3)	0.0523 (3)	0.0402 (3)	0.0071 (3)	0.0074 (2)	0.0088 (3)
Cl1	0.0811 (9)	0.1224 (11)	0.0619 (7)	0.0147 (8)	0.0189 (6)	−0.0027 (7)
Cl2	0.1116 (11)	0.0920 (9)	0.0594 (7)	0.0173 (8)	0.0104 (7)	−0.0025 (6)
Cl3	0.0801 (8)	0.1053 (10)	0.0563 (7)	−0.0014 (7)	0.0202 (6)	−0.0076 (7)
N1	0.043 (2)	0.057 (2)	0.050 (2)	0.0085 (17)	0.0069 (16)	0.0060 (17)
N2	0.0364 (19)	0.060 (2)	0.050 (2)	0.0120 (16)	0.0093 (16)	0.0124 (18)
N3	0.046 (2)	0.089 (3)	0.051 (2)	0.0173 (19)	0.0071 (18)	0.012 (2)
N4	0.040 (2)	0.072 (2)	0.052 (2)	0.0103 (18)	0.0118 (17)	0.0165 (18)
N5	0.0368 (19)	0.060 (2)	0.048 (2)	0.0127 (17)	0.0119 (17)	0.0190 (17)
N6	0.050 (2)	0.057 (2)	0.059 (2)	0.0136 (18)	0.0077 (18)	0.0059 (18)
N7	0.047 (2)	0.057 (2)	0.062 (2)	0.0009 (18)	0.0069 (18)	0.0117 (19)
N8	0.0355 (19)	0.046 (2)	0.051 (2)	0.0050 (16)	0.0086 (16)	0.0118 (16)
N9	0.0387 (19)	0.052 (2)	0.046 (2)	0.0069 (16)	0.0086 (16)	0.0082 (17)
N10	0.0346 (19)	0.054 (2)	0.0464 (19)	0.0113 (16)	0.0056 (15)	0.0086 (17)
N11	0.0306 (18)	0.0447 (19)	0.0417 (18)	0.0053 (15)	0.0082 (15)	0.0103 (15)
O1	0.0296 (14)	0.0511 (17)	0.0322 (14)	0.0038 (13)	0.0064 (12)	0.0007 (12)
O2	0.0385 (16)	0.0649 (19)	0.0454 (17)	−0.0036 (15)	0.0167 (14)	0.0044 (15)
O3	0.0391 (16)	0.0647 (19)	0.0468 (17)	0.0134 (15)	0.0049 (15)	0.0081 (15)
S1	0.0502 (7)	0.0720 (8)	0.0539 (6)	0.0008 (6)	0.0134 (5)	−0.0062 (6)
S2	0.0481 (7)	0.0740 (8)	0.0474 (6)	−0.0018 (6)	0.0103 (5)	−0.0020 (6)
C1	0.024 (2)	0.050 (3)	0.052 (2)	0.0080 (18)	0.0129 (18)	0.014 (2)
C2	0.026 (2)	0.048 (3)	0.051 (2)	0.0037 (18)	0.0081 (19)	0.009 (2)
C3	0.037 (2)	0.066 (3)	0.060 (3)	0.005 (2)	0.014 (2)	0.022 (2)
C4	0.036 (3)	0.095 (4)	0.060 (3)	0.004 (2)	0.005 (2)	0.019 (3)
C5	0.042 (2)	0.060 (3)	0.046 (2)	0.014 (2)	0.011 (2)	0.019 (2)
C6	0.049 (3)	0.092 (3)	0.055 (3)	−0.002 (2)	0.013 (2)	0.015 (3)
C7	0.055 (3)	0.116 (4)	0.056 (3)	0.007 (3)	0.020 (2)	0.017 (3)
C8	0.060 (3)	0.068 (3)	0.053 (3)	0.016 (2)	0.016 (2)	0.013 (2)
C9	0.048 (3)	0.075 (3)	0.062 (3)	−0.002 (2)	0.014 (2)	0.008 (2)
C10	0.046 (3)	0.077 (3)	0.060 (3)	0.002 (2)	0.016 (2)	0.012 (2)
C11	0.038 (3)	0.060 (3)	0.065 (3)	0.001 (2)	0.013 (2)	0.014 (2)
C12	0.043 (2)	0.066 (3)	0.064 (3)	0.012 (2)	0.015 (2)	0.006 (2)
C13	0.042 (2)	0.046 (2)	0.047 (2)	0.010 (2)	0.011 (2)	0.018 (2)
C14	0.048 (3)	0.062 (3)	0.070 (3)	0.008 (2)	0.019 (2)	0.021 (2)
C15	0.070 (3)	0.072 (3)	0.057 (3)	0.013 (3)	0.024 (2)	0.012 (2)
C16	0.070 (3)	0.054 (3)	0.045 (3)	0.011 (2)	0.008 (2)	0.009 (2)
C17	0.059 (3)	0.069 (3)	0.057 (3)	−0.008 (2)	0.009 (2)	0.002 (2)
C18	0.056 (3)	0.077 (3)	0.060 (3)	−0.002 (2)	0.022 (2)	0.015 (2)
C19	0.027 (2)	0.062 (3)	0.052 (2)	0.004 (2)	0.0068 (19)	0.016 (2)
C20	0.034 (2)	0.060 (3)	0.055 (3)	0.010 (2)	0.018 (2)	0.010 (2)
C21	0.034 (2)	0.046 (2)	0.045 (2)	0.0076 (18)	0.0099 (18)	0.0128 (19)
C22	0.035 (2)	0.066 (3)	0.050 (3)	0.002 (2)	0.011 (2)	0.008 (2)
C23	0.047 (3)	0.072 (3)	0.059 (3)	0.012 (2)	0.023 (2)	0.013 (2)
C24	0.063 (3)	0.055 (3)	0.046 (3)	0.007 (2)	0.013 (2)	0.007 (2)
C25	0.044 (3)	0.068 (3)	0.054 (3)	0.001 (2)	0.011 (2)	0.003 (2)
C26	0.036 (2)	0.065 (3)	0.053 (3)	0.009 (2)	0.016 (2)	0.008 (2)

### Geometric parameters (Å, °)

**Table:**

Fe1—N1	2.086 (3)	S2—C2	1.633 (4)
Fe1—O2	2.100 (2)	C3—H7	0.9300
Fe1—O3	2.102 (3)	C4—H8	0.9300
Fe1—N2	2.107 (3)	C5—C10	1.378 (4)
Fe1—O1^i^	2.264 (3)	C5—C6	1.387 (4)
Fe1—O1	2.281 (2)	C6—C7	1.383 (5)
Cl1—C8	1.752 (4)	C6—H9	0.9300
Cl2—C16	1.738 (4)	C7—C8	1.362 (5)
Cl3—C24	1.745 (4)	C7—H10	0.9300
N1—C1	1.169 (4)	C8—C9	1.373 (4)
N2—C2	1.162 (4)	C9—C10	1.372 (4)
N3—C4	1.285 (4)	C9—H11	0.9300
N3—N4	1.376 (4)	C10—H12	0.9300
N4—C3	1.305 (4)	C11—H13	0.9300
N5—C4	1.351 (4)	C12—H14	0.9300
N5—C3	1.360 (4)	C13—C14	1.377 (4)
N5—C5	1.432 (4)	C13—C18	1.384 (4)
N6—C12	1.306 (4)	C14—C15	1.380 (4)
N6—N7	1.397 (4)	C14—H15	0.9300
N7—C11	1.298 (4)	C15—C16	1.357 (5)
N8—C12	1.352 (4)	C15—H16	0.9300
N8—C11	1.359 (4)	C16—C17	1.370 (4)
N8—C13	1.428 (4)	C17—C18	1.378 (4)
N9—C20	1.300 (4)	C17—H17	0.9300
N9—N10	1.381 (3)	C18—H18	0.9300
N10—C19	1.296 (4)	C19—H19	0.9300
N11—C19	1.361 (4)	C20—H20	0.9300
N11—C20	1.359 (4)	C21—C26	1.387 (4)
N11—C21	1.429 (4)	C21—C22	1.387 (4)
O1—Fe1^i^	2.264 (3)	C22—C23	1.374 (4)
O1—H1	0.869 (10)	C22—H21	0.9300
O1—H2	0.880 (10)	C23—C24	1.371 (4)
O2—H3	0.884 (10)	C23—H22	0.9300
O2—H4	0.881 (10)	C24—C25	1.372 (4)
O3—H5	0.885 (10)	C25—C26	1.375 (4)
O3—H6	0.875 (10)	C25—H23	0.9300
S1—C1	1.632 (4)	C26—H24	0.9300
			
N1—Fe1—O2	90.22 (11)	C8—C7—H10	120.3
N1—Fe1—O3	89.68 (12)	C6—C7—H10	120.3
O2—Fe1—O3	101.01 (10)	C7—C8—C9	121.6 (4)
N1—Fe1—N2	178.33 (12)	C7—C8—Cl1	120.2 (3)
O2—Fe1—N2	89.47 (11)	C9—C8—Cl1	118.2 (3)
O3—Fe1—N2	88.76 (11)	C8—C9—C10	119.0 (4)
N1—Fe1—O1^i^	90.32 (11)	C8—C9—H11	120.5
O2—Fe1—O1^i^	89.04 (10)	C10—C9—H11	120.5
O3—Fe1—O1^i^	169.95 (9)	C9—C10—C5	120.7 (4)
N2—Fe1—O1^i^	91.32 (11)	C9—C10—H12	119.6
N1—Fe1—O1	89.79 (10)	C5—C10—H12	119.6
O2—Fe1—O1	167.40 (10)	N7—C11—N8	111.9 (3)
O3—Fe1—O1	91.59 (10)	N7—C11—H13	124.0
N2—Fe1—O1	90.87 (10)	N8—C11—H13	124.0
O1^i^—Fe1—O1	78.36 (9)	N6—C12—N8	111.7 (3)
C1—N1—Fe1	175.9 (3)	N6—C12—H14	124.2
C2—N2—Fe1	172.4 (3)	N8—C12—H14	124.2
C4—N3—N4	107.0 (3)	C14—C13—C18	119.2 (4)
C3—N4—N3	105.8 (3)	C14—C13—N8	120.7 (3)
C4—N5—C3	102.1 (3)	C18—C13—N8	120.1 (3)
C4—N5—C5	129.6 (3)	C15—C14—C13	120.4 (4)
C3—N5—C5	128.4 (3)	C15—C14—H15	119.8
C12—N6—N7	106.4 (3)	C13—C14—H15	119.8
C11—N7—N6	106.3 (3)	C16—C15—C14	120.1 (4)
C12—N8—C11	103.7 (3)	C16—C15—H16	120.0
C12—N8—C13	128.3 (3)	C14—C15—H16	120.0
C11—N8—C13	127.9 (3)	C15—C16—C17	120.2 (4)
C20—N9—N10	106.8 (3)	C15—C16—Cl2	120.3 (3)
C19—N10—N9	106.9 (3)	C17—C16—Cl2	119.4 (3)
C19—N11—C20	103.5 (3)	C16—C17—C18	120.3 (4)
C19—N11—C21	128.0 (3)	C16—C17—H17	119.8
C20—N11—C21	128.5 (3)	C18—C17—H17	119.8
Fe1^i^—O1—Fe1	101.64 (9)	C17—C18—C13	119.7 (4)
Fe1^i^—O1—H1	104 (3)	C17—C18—H18	120.1
Fe1—O1—H1	119 (2)	C13—C18—H18	120.1
Fe1^i^—O1—H2	110 (3)	N10—C19—N11	111.4 (3)
Fe1—O1—H2	114 (2)	N10—C19—H19	124.3
H1—O1—H2	108 (2)	N11—C19—H19	124.3
Fe1—O2—H3	128 (2)	N9—C20—N11	111.3 (3)
Fe1—O2—H4	125 (2)	N9—C20—H20	124.3
H3—O2—H4	106 (2)	N11—C20—H20	124.3
Fe1—O3—H5	119 (3)	C26—C21—C22	119.0 (3)
Fe1—O3—H6	117 (2)	C26—C21—N11	120.8 (3)
H5—O3—H6	106 (2)	C22—C21—N11	120.2 (3)
N1—C1—S1	179.6 (3)	C23—C22—C21	120.1 (3)
N2—C2—S2	179.7 (3)	C23—C22—H21	119.9
N4—C3—N5	112.2 (3)	C21—C22—H21	119.9
N4—C3—H7	123.9	C24—C23—C22	120.2 (4)
N5—C3—H7	123.9	C24—C23—H22	119.9
N3—C4—N5	112.9 (4)	C22—C23—H22	119.9
N3—C4—H8	123.6	C25—C24—C23	120.4 (4)
N5—C4—H8	123.6	C25—C24—Cl3	119.9 (3)
C10—C5—C6	119.4 (4)	C23—C24—Cl3	119.7 (3)
C10—C5—N5	120.8 (3)	C24—C25—C26	119.8 (4)
C6—C5—N5	119.8 (3)	C24—C25—H23	120.1
C5—C6—C7	119.9 (4)	C26—C25—H23	120.1
C5—C6—H9	120.1	C25—C26—C21	120.4 (3)
C7—C6—H9	120.1	C25—C26—H24	119.8
C8—C7—C6	119.4 (4)	C21—C26—H24	119.8

Symmetry codes: (i) −*x*+1, −*y*, −*z*+1.

### Hydrogen-bond geometry (Å, °)

**Table:**

*D*—H···*A*	*D*—H	H···*A*	*D*···*A*	*D*—H···*A*
O1—H1···N10^ii^	0.87 (3)	1.97 (3)	2.827 (4)	170 (3)
O1—H2···N9	0.88 (3)	1.94 (3)	2.819 (4)	173 (3)
O2—H3···N7^iii^	0.88 (3)	1.98 (3)	2.866 (5)	178 (3)
O2—H4···N4^iv^	0.88 (2)	1.97 (3)	2.853 (4)	175 (3)
O3—H5···N6	0.88 (3)	1.92 (3)	2.802 (4)	174 (3)
O3—H6···N3^v^	0.88 (2)	1.93 (2)	2.803 (4)	172 (3)
C3—H7···S2^vi^	0.93	2.72	3.624 (5)	165
C22—H21···S2^ii^	0.93	2.87	3.736 (5)	156
C11—H13···S1^vii^	0.93	2.87	3.783 (5)	167

Symmetry codes: (ii) −*x*, −*y*, −*z*+1; (iii) *x*+1, *y*, *z*; (iv) *x*, *y*−1, *z*; (v) *x*−1, *y*−1, *z*; (vi) *x*, *y*+1, *z*; (vii) *x*−1, *y*, *z*.
